# Exploration and Development of a Simpler Respiratory Distress Observation Scale (modRDOS-4) as a Dyspnea Screening Tool: A Prospective Bedside Study

**DOI:** 10.1089/pmr.2020.0094

**Published:** 2021-01-06

**Authors:** Ru Xin Wong, Ho Shirlynn, Yen Sin Koh, Stella Goh Seow Lin, Daniel Quah, Qingyuan Zhuang

**Affiliations:** ^1^ Division of Radiation Oncology, National Cancer Centre Singapore, Singapore, Singapore.; ^2^ Division of Supportive and Palliative Care, National Cancer Centre Singapore, Singapore, Singapore.

**Keywords:** breathless, dyspnea, end of life, nursing, palliative, symptom control

## Abstract

***Introduction:*** End-of-life patients face difficulties in reporting respiratory distress. The Respiratory Distress Observation Scale (RDOS) is a well-known tool; however, field implementation has been challenging from ground feedback. We sought to develop a simpler scale.

***Setting:*** Patients referred for palliative consult in a tertiary hospital in Singapore were recruited.

***Methods:*** *A priori*, we identified 18 dyspnea physical signs and documented their presence through bedside observation. Dyspnea severity was self-reported. The cohort was randomly split into training and test sets. Partial least square regression with leave-one-out cross-validation was used to develop a four-point model from the training set. Using the test set, data fit was compared using Akaike and Bayesian Information Criterion. Discrimination was assessed using receiver operating characteristics.

***Results:*** Of 122 patients, mean age was 67.9 years (range 23–93, standard deviation 12.9), 71.3% had a primary cancer diagnosis, and 58.1% were chair/bedbound with a Palliative Performance Scale of ≤50. Median reported dyspnea scale was 5 (interquartile range 3–7). Our model (modRDOS-4) consisted of four predictors (grunting, respiratory rate, accessory muscle use, paradoxical breathing). A modRDOS-4 of ≥6 identified moderate-to-severe dyspnea with a sensitivity of 0.78 and specificity of 0.90. Using the test set, with the modRDOS-4, the Akaike Information Criterion (AIC) is 149.8, Bayesian Information Criteria (BIC) is 154.1, and the receiver operating characteristics (ROC) is 0.74. With the original RDOS, the AIC is 145.2, BIC is 149.5, and ROC is 0.76.

***Conclusion:*** For a quick assessment of dyspnea, we developed a four-item tool with a pilot web-based nomogram. External validation is needed.

## Introduction

Dyspnea is common at the end of life (EOL), and can happen due to varied conditions, for example, pulmonary neoplasms; end-stage pulmonary disease; renal, liver, and cardiac disorders; and pneumonia.^1,2^ Patient-reported dyspnea is the gold standard for assessment, but in uncommunicative or cognitively impaired patients, a surrogate measure is needed. Many EOL patients have declining consciousness and/or cognitive function and may not be able to accurately report dyspnea.^3^

The Respiratory Distress Observation Scale (RDOS) was developed by Campbell et al.^4^ from a biobehavioral framework and is the most well-known tool for assessing dyspnea in uncommunicative EOL patients. Cutoff points suggesting mild, moderate, and severe respiratory distress have also been determined to enhance clinical utility.^5^ Other groups have adapted it for use in the intensive care unit (ICU) setting in patients on mechanical ventilators.^6,7^ We had previously validated the RDOS in 122 palliative care patients within a tertiary hospital and found it to be valid and reliable.^8^ In addition, as a secondary aim to find out the prevalence of physical signs of dyspnea among this population, we developed a list of 18 physical signs of dyspnea and administered it to this cohort simultaneously with the validation study.

Despite the successful validation of RDOS, we faced considerable challenges in implementation among general ward nurses in our hospital. Palliative care patients reside in different wards and attending nurses are of varying seniority and experience with EOL patients. Some users feedback that the scale was burdensome, in comparison with routinely used scales such as the Richmond Agitation Sedation Scale (RASS),^9^ Glasgow Coma Scale (GCS),^10^ and Critical Care Pain Observation Tool (CPOT),^11^ which are considerably shorter. Some users expressed uncertainties in scoring the subjective items such as restlessness and look of fear, which are awarded two points each in the RDOS. Because the cutoff scoring of ≥4 suggests moderate-to-severe respiratory distress necessitating intervention, a decision regarding presence or absence of these subjective items would influence the score going below or above the cutoff with a direct impact on management.

Thus, we aimed to develop a simpler scale by conducting a secondary analysis on our previous dataset of dyspnea physical signs. In developing this new scale, we sought to balance the need for low item burden and objectivity against scale accuracy. Our hypothesis is that a simplified scale with an electronic interface would improve clinical utilization and uptake in hospitals, hospices, and nursing homes as a dyspnea screening tool.

## Methods

### Study setting

In our parent RDOS validation study,^8^ palliative care patients (*n* = 122) were recruited from a single 1597-bed tertiary hospital with a dedicated palliative care team that provides consultation services.

We included patients with age ≥21 years, at risk of dyspnea (but not referred specifically for management of dyspnea) with one of these diagnoses: malignancies in the lung or pleura, renal failure, heart failure, and chronic obstructive pulmonary disease (COPD). We also included patients referred specifically for management of dyspnea. Additional details on the population studied are available in the original validation article.^8^

### Methods and measures

*A priori*, we identified 18 physical signs known to be associated with dyspnea. An informal discussion with accredited palliative physicians and respiratory specialists working in a tertiary institution was done to come up with these 18 respiratory signs. Eight signs were from the original RDOS and 10 additional signs were identified after literature review^12^ and collecting input from palliative and respiratory specialists (Table 1). These signs were derived from teachings in medical/nursing school and physical examination textbooks.^13^

**Table 1.** List of 18 *a Priori* Respiratory Signs/Features Associated with Dyspnea

Original RDOS	
1	Heart rate/minute
2	Respiratory rate/minute
3	Restlessness
4	Paradoxical breathing pattern
5	Accessory muscle use
6	Grunting at end expiration
7	Nasal flaring
8	Look of fear
Additional 10 signs	
1	Intercostal retraction
2	Subcostal retraction
3	Substernal retraction
4	Suprasternal retraction
5	Tripod positioning
6	Stridor
7	Wheeze
8	Pursed lips breathing
9	Diaphoresis
10	Requiring supplemental oxygen

RDOS, Respiratory Distress Observation Scale.

We documented the presence of these physical signs through bedside observation.

The reference standard for dyspnea measurement is self-report. We utilized two single-item verbal report scales to assess the patient's current dyspnea severity. The Dyspnea Numeric Rating Scale (dyspnea-NRS) is a widely used and a valid scale to measure severity. It is scored from 0 to 10, labeled with verbal anchors (e.g., “nothing at all” to “maximal”).^14^ The Dyspnea Categorical Verbal Descriptor Scale (dyspnea-Cat) is a four-level categorical scale to describe severity of dyspnea (none, mild, moderate, and severe). It has been shown to have strong correlation with the dyspnea-NRS.^15^

### Procedure

Inpatient referrals were screened daily and patients who met the inclusion criteria were identified. After obtaining verbal consent, the rater would proceed to administer the RDOS. After administering the RDOS, the rater would then administer our list of 18 physical signs of dyspnea. Lastly, this would be followed by obtaining patient self-report on dyspnea using the dyspnea-NRS and dyspnea-Cat.

### Statistical analysis

One hundred twenty-two patients from the original cohort used to validate the original RDOS were randomly split into training and test sets (ratio 3:1). Within the training set, univariate analysis was used to determine the symptoms that were significantly associated (*p* < 0.05) with the dyspnea-NRS. The significant symptoms were selected and partial least square (PLS) regression with leave-one-out cross-validation (LOOCV) was used to rank the variables to develop the model.

PLS is a method for constructing predictive models when the factors are many and highly collinear.^16^ LOOCV is a type of cross-validation method to determine the predictive ability of a model using an internal “train” dataset.

The eventual scale was derived using a regression coefficient-based scoring system.

Using the test set, comparisons between the new scale were made with the original RDOS.

Scale discrimination for dyspnea-Cat severity was assessed using receiver operating characteristics (ROC). ROC is a popular tool to assess the discrimination ability of a score with a binary outcome classifier, in this case the presence of at least moderate dyspnea or not. A higher area under the curve means better discrimination.^17^

Data fit was assessed using Akaike Information Criterion (AIC) and Bayesian Information Criteria (BIC). Lower values mean the model is closer to the truth.^18^

A suggested cutoff to predict for moderate-to-severe dyspnea was determined with Youden's Index, which is a function of sensitivity and specificity to obtain a model threshold.^19^

Statistical analysis was performed using RStudio [RStudio Team (2015) Boston, MA; www.rstudio.com] and the nomogram was developed and hosted with Shiny.

### Ethics approval

Approval to conduct the study was granted by the Singhealth Institutional Review Board (CIRB Ref No.: 2017/3126) with waiver of written informed consent as the study presented minimal risk of harm to participants.

## Results

Of 122 patients, mean age was 67.9 (range 23–93, standard deviation 12.9), 71.3% had a primary cancer diagnosis, and 58.1% were chair-/bedbound with a Palliative Performance Scale of ≤50. Median reported dyspnea scale was 5 (interquartile range 3–7) (Table 2).

**Table 2.** Baseline Characteristics of Study Participants

Variable	*N* = 122
Age (years), mean (SD)	67.9 (12.9)
Gender (%)	
Male	69 (56.6)
Female	53 (43.4)
Race (%)	
Chinese	99 (81.1)
Malay	14 (11.5)
Indian	9 (7.4)
Others	0 (0)
Primary diagnosis (%)	
Lung or pleural malignancies	41 (33.6)
End-stage renal failure	12 (9.8)
Heart failure	17 (13.9)
Chronic obstructive pulmonary disease	5 (4.1)
Others	47 (38.5)
Palliative Performance Scale (%)	
100	0 (0)
90	1 (0.8)
80	6 (4.9)
70	19 (15.6)
60	25 (20.5)
50	28 (23.0)
40	15 (12.3)
30	15 (12.3)
20	13 (10.7)
10	0 (0)
Median RDOS score (range)^a^	3 (0–10)
Dyspnea category^b^	
None	22 (18.0)
Mild	36 (29.5)
Moderate	45 (36.9)
Severe	19 (15.6)

^a^ RDOS measures respiratory distress on a scale of 0 to 10.

^b^ Dyspnea-category: Dyspnea Categorical Verbal Descriptor Scale is a four-level categorical scale to describe severity of dyspnea.

SD, standard deviation.

### Train set

**FIG. 1.**
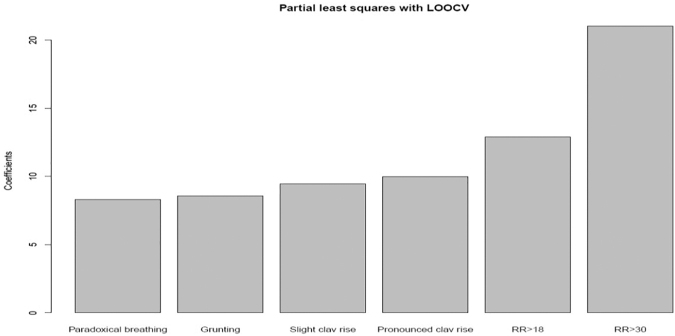
The coefficients of the four points that were developed with partial least square method. clav, clavicle; RR, respiratory rate.

Using the training set of 92 patients, out of the 18 *a priori* chosen symptoms, those that were significantly associated with rise in dyspnea-NRS scores were retractions, pursed lips, tachycardia, respiratory rate, restlessness, paradoxical breathing, accessory muscle use, grunting, nasal flaring, and look of fear. Using PLS regression with LOOCV, these 10 variables were ranked. We examined the ranked variables and noted that restlessness was the fifth variable. Owing to end-user feedback that restlessness was difficult to score, we decided to only incorporate the top-ranked four points. The top four symptoms were grunting, respiratory rate, accessory muscle use (clavicle rise), and paradoxical breathing (GRAP) (Fig. 1). See Supplementary Table S1 for ranked list of variables from PLS regression.

Using multivariable regression and after rounding the regression coefficients up to the nearest 0.5, the eventual equation is:

Dyspnea numerical rating scale = 2.0 (18 < respiratory rate >30) or 2.5 (respiratory rate ≥30) + 2.0 (slight clavicle rise) or 4.0 (pronounced clavicle rise) +1.5 (grunting present) +1.0 (paradoxical breathing) (see Table 3).

**Table 3.** Variables in modRDOS-4 (GRAP) and Corresponding Points

Point Variable	0	1	1.5	2	2.5	4	Total
Grunting	Absent		Present				≥6
Respiratory rate	≤18			>18	>30		(78% sensitivity and 90% specificity to detect moderate-to-severe dyspnea)
Accessory muscle (clavicle rise)	None			Slight		Pronounced	≥4
Paradoxical breathing	Absent	Present					(98% sensitivity, 43% specificity)

A user-friendly electronic interface is made available on breathless.shinyapps.io/GRAP/ (Supplementary Figure S1).

GRAP, grunting, respiratory rate, accessory muscle use, paradoxical breathing.

As these four symptoms were originally in the RDOS, we decided to name this modified-RDOS (modRDOS-4).

**FIG. 2.**
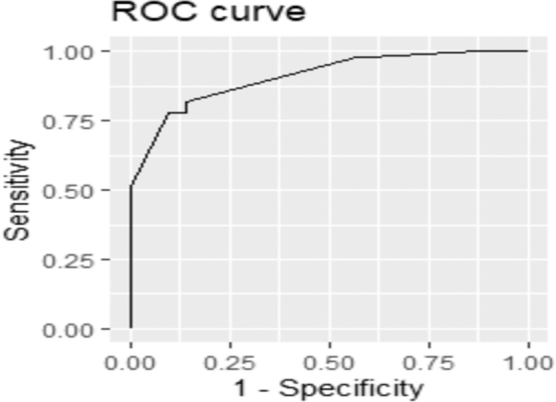
ROC using modRDOS-4 for moderate/severe dyspnea. modRDOS-4; ROC, receiver operating curve.

**FIG. 3.**
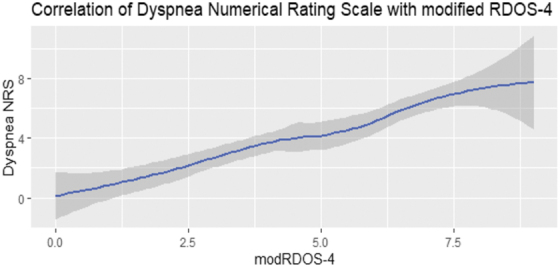
Correlation of mRDOS-4 with dyspnea numerical rating scale.

The modRDOS-4 showed good discrimination with an ROC of 0.91 (Fig. 2) and good correlation between modRDOS-4 scores and dyspnea-NRS scores (*r* = 0.73) (Fig. 3).

Using Youden's Index, we suggest a modRDOS-4 score cut-off of ≥6 to predict for moderate-to-severe dyspnea with a sensitivity of 78% and specificity of 90%. When the cost ratio of false negative to false positive was set to 10:1, a suggested cut-off points of ≥4 would predict for moderate-to-severe dyspnea with a sensitivity of 98% and specificity of 43%.

### Test set

We subsequently validated the modRDOS-4 on our test set of 30 patients and obtained an ROC of 0.74, AIC of 149.8, and BIC of 154.1. The original RDOS has an ROC of 0.76, AIC of 145.2, and BIC of 149.5 on the same test set.

## Discussion

The modRDOS-4 is more parsimonious and has somewhat similar discrimination and fit compared with the original RDOS. We propose the modRDOS-4 as a tool that can be easily adopted within different EOL settings. Features of this shortened tool include reduced burden of administration, less training requirement, and utilization of a simple mnemonic (GRAP). We have also developed a pilot online interface to improve ease of access and hopefully improve adoption. Our modRDOS-4 with the acronym GRAP can be found at https://breathless.shinyapps.io/GRAP/ (Supplementary Figure S1) although this is not to be implemented until further external validation. The site can be saved to any smart phone home screen and used as an application. We have added in a short tutorial in the site too. We hope that after more external validation, this tool may be useful even within the home hospice setting to provide caregivers the ability to make adequate assessment of dyspnea. The original RDOS remains the gold standard in experienced and trained hands.

The threshold of the modRDOS-4 can be changed by factoring the cost of false positive or negative differently. For example, to increase the sensitivity of the tool to ∼100%, a cut-off of 4 is used but at the cost of decreased specificity. This may be useful in a situation that requires an initial screening to determine whether further assessment of dyspnea is necessary.

In developing the modified model, adding the fifth ranked variable “restlessness” to the model would have improved its properties. However, we decided to remove it as prior bedside experience using the original RDOS suggested to us that “restlessness” was a variable that was difficult to assess and had significant inter-rater variability. Hence the eventual model had only four variables. Theoretically, we feel that the four points (GRAP) represents different physiological explanations for dyspnea. Grunting is an attempt to maintain positive airway pressure.^20^ Respiratory rate is a physiologic response to hypercapnia.^21^ Activation of accessory muscle usage is due to increased work of breathing. Paradoxical breathing is a phenomenon due to a fatigued diaphragm.^22^ These four points are also within the original RDOS that was developed using a behavioral model^23^ and from observation of patients who were weaned off ventilator.^24^

There are limitations to the modRDOS-4. The same considerations in using the original RDOS applies to the modRDOS-4. Both tools are meant to be used in adults and cannot be used in patients with neuromuscular disorders. It has not been externally validated. Our decision to have four points is also arbitrary due to the deliberate omission of the fifth ranked variable, restlessness. We acknowledge that the modRDOS-4 has no variable to capture the patient's affective response to respiratory distress, which may diminish its construct validity since dyspnea is a multidimensional symptom. However, this allows us to achieve our aim in designing a rapid and easy-to-use screening tool for respiratory distress in uncommunicative patients. Titration of medications (such as opioids) for management of respiratory distress should still be guided by more comprehensive assessment such as the RDOS.

In addition, in our personal communication with Dr Margaret Campbell, she shared that the uptake and implementation of the original RDOS in her country are without difficulty, contrary to our personal experience. Further work to ascertain temporal trajectory of modRDOS-4 and changes after treatment of dyspnea are necessary too.

## Conclusion

For an easier assessment of respiratory distress, we developed and internally validated a four-item tool with a pilot web-based nomogram. It may be a quicker and easier tool to screen for dyspnea in uncommunicative patients, especially in a community setting. We hope to invite further external validation of this tool. The original RDOS remains the gold standard to assess dyspnea in an uncommunicative patient.

## Dataset Sharing

The anonymized dataset of patients' symptoms can be made available upon request to the authors.

## Supplementary Material

Supplemental data

Supplemental data

## Abbreviations Used

AIC: Akaike Information Criterion
BIC: Bayesian Information Criteria
dyspnea-Cat: Dyspnea Categorical Verbal Descriptor Scale
dyspnea-NRS: Dyspnea Numeric Rating Scale
EOL: end of life
GRAP: grunting, respiratory rate, accessory muscle use, paradoxical breathing
LOOCV: leave-one-out cross-validation
PLS: partial least square
RDOS: Respiratory Distress Observation Scale
ROC: receiver operating characteristics
SD: standard deviation
