# Supplementary material for: Exploration and Development of a Simpler Respiratory Distress Observation Scale (modRDOS-4) as a Dyspnea Screening Tool: A Prospective Bedside Study
Source: Palliat Med Rep. 2021 Jan 6;2(1):9–14. doi: 10.1089/pmr.2020.0094 (PMC8241376; doi:10.1089/pmr.2020.0094)
Supplement: Supplemental data [file Supp_Table1.docx]

| Supplement table: List of respiratory signs and coefficients from Partial Least Square Regression | |
| --- | --- |
| Signs | Coefficients |
| Retractions | -7.413610635 |
| Pursed lip | 0.1370264866 |
| Tripod positioning | 2.547862547 |
| Nasal flaring | 4.929002149 |
| Look of fear | 7.149255253 |
| Restlessness | 7.583126406 |
| Paradoxical breathing | 8.313638641 |
| Grunting | 8.569313186 |
| Clavicle rise, slight | 9.458092146 |
| Respiratory rate/min <30 | 9.979713865 |
| Respiratory rate/min =>30 | 12.9045728 |
| Clavicle rise, pronounced | 21.01478588 |
