# Supplementary figures and images for: Exploration and Development of a Simpler Respiratory Distress Observation Scale (modRDOS-4) as a Dyspnea Screening Tool: A Prospective Bedside Study
Source: Palliat Med Rep. 2021 Jan 6;2(1):9–14. doi: 10.1089/pmr.2020.0094 (PMC8241376; doi:10.1089/pmr.2020.0094)

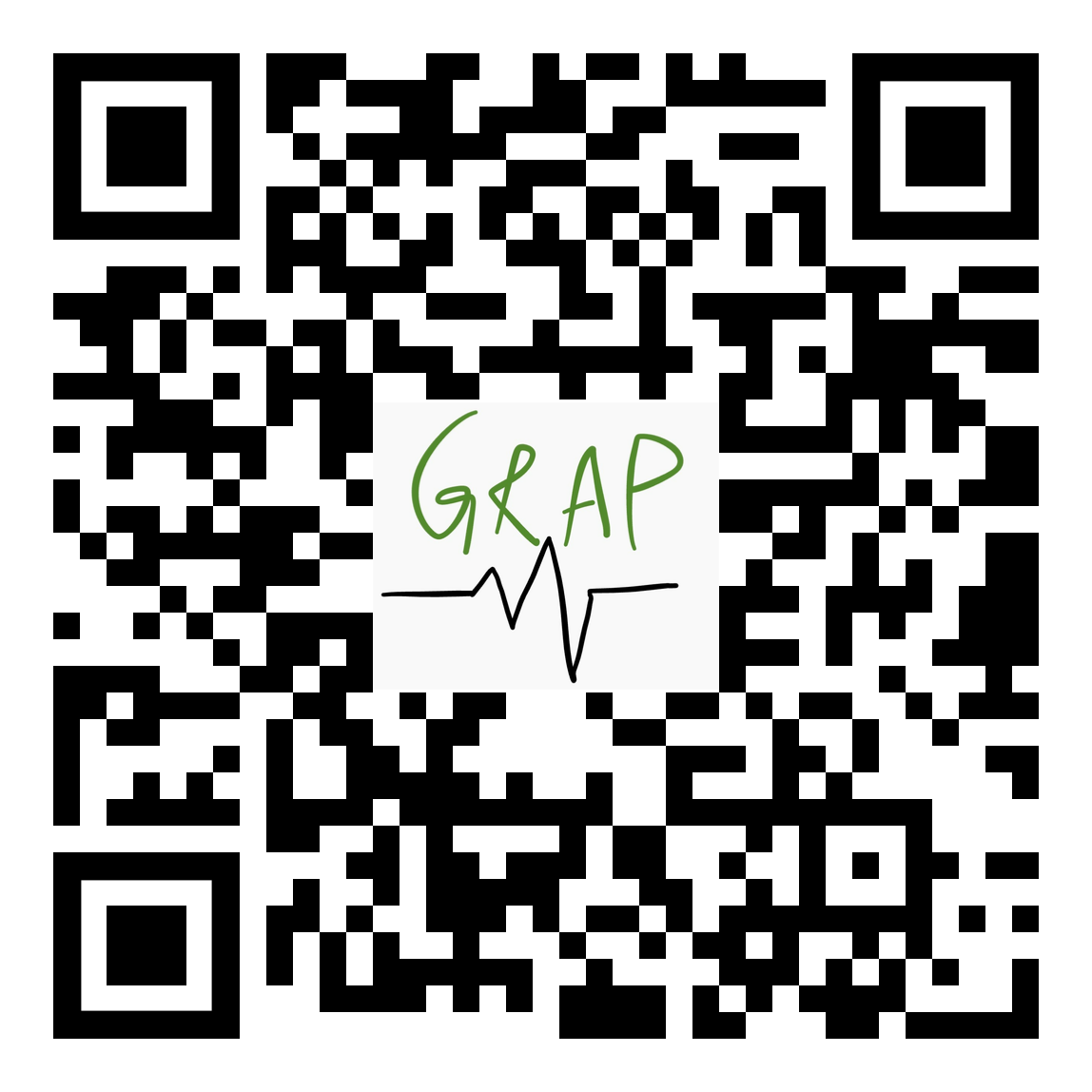

Supplement: Supplemental data [file Supp_Fig1.tiff]
